# Benefit packages for chronic disease outpatients in the New Rural Cooperative Medical Scheme in 32 Chinese counties

**DOI:** 10.12688/f1000research.2-137.v1

**Published:** 2013-06-10

**Authors:** Chuangzhou Xu, Christian A Gericke

**Affiliations:** 1 International Business School, Shaanxi Normal University, Yangling, Shaanxi, 710062, China; 2 The Wesley Research Institute, Brisbane, Queensland, QLD 4066, Australia; 3 University of Queensland School of Population Health, Brisbane, Queensland, QLD 4006, Australia; 4 Queensland University of Technology School of Public Health, Brisbane, Queensland, QLD 4059, Australia

## Abstract

**Introduction:** Chronic disease has become a major problem affecting the health of the Chinese population. In response to this situation, the New Rural Cooperative Medical Scheme (NRCMS) has begun to provide health cover for outpatients with chronic disease expenses, made possible by the increased risk pool of previous years. We compare the differences between Benefit Packages for Chronic Diseases Outpatients (BPCDO) in order to produce a reference for policy makers.

**Methods:** Information on the various BPCDO was located by searching the official NRCMS website in Chinese, using certain criteria to select the ideal BPCDO. Population coverage, service coverage and cost of coverage were chosen to form the analytical framework for this paper. The diseases were classified according to the World Health Organisation's (WHO) International Classification of Diseases (ICD-10).

**Results:** To avoid “moral hazard”, complex processes have been created. This has resulted in chronic disease patients finding it very difficult to become beneficiaries. Forty-one types of chronic diseases were listed in 32 different BPCDO. We found that different counties have different co-payment rates, deductible lines, ceilings, coverage of drugs and tests, appointed hospitals and reimbursement frequencies.

**Conclusion:** High mortality diseases and diseases with a heavier cost burden should be the priority on the list of reimbursement. The BPCDO scheme should be introduced urgently at the national level. It should include twenty-one types of disease and eight essential factors.

## Introduction

China is becoming an ageing society and, along with the changing social economy, high-risk unhealthy behaviors have increased. Among the high risk behaviors, an increase in dietary fat and salt intake, reduced physical activity, and high rates of smoking in the male population are all long standing issues ^1^. All of these factors have contributed to the increasing number and proportion of patients suffering from chronic disease ^1, 2^. The fourth report of the National Health Services Survey in 2008 ^3^, reported that 20% of the Chinese population were living with a chronic disease, an increase of 4.9% since 2003. The report also highlighted the increasingly higher proportion of rural residents living with a chronic disease. The survey estimated that nationally the total number of chronic disease cases reached 260 million in 2008, with rural residents accounting for 163 million ^3^.

Chronic disease accounted for almost 80% of all deaths, and 70% of the total disability adjusted life years (DALY) lost in 2005 ^2^. The challenges caused by chronic diseases are predicted to increase in severity in the foreseeable future ^4, 5^. Unfortunately, China has performed poorly in addressing non-communicable diseases ^6^, and rural areas have experienced the heaviest burden of chronic disease ^7^.

Since the implementation of the New Rural Cooperative Medical Scheme (NRCMS) in 2003, the main goal has been to reduce the burden of medical expenses, in particular, for farmers who are inpatients with catastrophic illnesses, for which expenses are very high. Although the NRCMS was initially incapable of covering outpatients with chronic diseases due to limited funds, in recent years the minimum funds for each farmer have increased from 30 Yuan in 2003 ^8^ to 50 Yuan in 2007 ^9^, and to 100 Yuan in 2009 ^10^ (US$1≈6.3 Yuan, Sept 2012). This increased financing has meant that the scheme now has the ability to cover some chronic disease expenses for outpatients, as these expensive outpatient services for are one of the main causes of medical impoverishment ^11^. Reimbursement for chronic disease treatment is a beneficial supplement to the NRCMS’s main achievements.

The NRCMS is currently organized at county level, resulting in relatively independent policy-making, with each county being responsible for deciding what is contained within its own benefits package. Research from other transition countries strongly suggests that diseases that are the major causes of ill-health should be included in community-based health insurance ^12^. From 2006, a few counties started to provide reimbursement to outpatients with chronic diseases, with an increasing number of counties offering reimbursement from 2008 on. Certain provinces, including Anhui, Yunan and Guangxi, have introduced a new policy in order to provide guidelines for the reimbursement of chronic disease expenses; however, not enough detail is included to assist with its uptake. There is still no national policy at present, and, as for research on NRCMS benefit packages, much of the focus is on inpatients ^13– 15^, and there are only few papers related to designing Benefit Packages for Chronic Diseases Outpatients (BPCDO) ^16, 17^. Our research compares the differences between the BPCDO offered in different counties in China, in order to measure existing inequalities and to produce a point of reference for national and county government policy decision makers.

Benefit packages aim to focus scarce resources on interventions where they are most needed ^18^. Benefit package design needs to encompass the scope of coverage and include the selection of services and providers, and the beneficiary obligations ^19^. Finiteness is a basic characteristic of any benefit package; it cannot include everything, so identifying the priority medical services is the most important process step ^20^. Some benefit packages are designed for particular populations or disease groups, such as HIV/AIDS prevention or maternal health interventions ^18^.

## Methods

### Benefit packages selection

The BPCDO we used were found on official websites. Two steps were used when selecting the BPCDO.

Step one: in order to select a representative sample, the geography, number of counties in each area and their economic development were taken into consideration. According to the administrative divisions of China, the whole country has 2,860 counties distributed into Eastern (697), Central (1086) and Western regions (1077) ^21^. According to Chinese National Bureau of Statistics criteria, there are also 592 poverty-stricken counties located in the Eastern (72), Central (204) and Western regions (316). Each part of China also has its own Top One Hundred Rich Counties. According to this information, we selected 32 counties as sample counties which were distributed in the Eastern (eight counties including one rich county and one poor county), Central (12 counties including one rich county and two poor counties) and Western regions (12 counties including one rich county and three poor counties) to model a representative sample of China’s rural areas.

Step two: in order to find the BPCDO statements in step one, we used the Google search input terms “cooperative medical schemes” and “chronic diseases” in Chinese. The selection of 32 plans was based on the following criteria: (1) The plan was on an official website, that is, a website of the NRCMS Office, Health Bureau or local government; (2) The final version of the plan was selected if more than one version was located for the same county; (3) Plans that did not mention the types of chronic disease and the amount of reimbursement were omitted; (4) No more than two counties from the same province were selected in order to avoid the inclusion of more than two counties with the same guidelines; (5) The plan was chosen if it met the demands of step one, until all plans required were obtained.

According to the above process, 32 plans (counties) were selected across 20 provinces between the 11 November and the 4 December 2009. The names, province, and economic development statuses of the selected counties are shown in Table 1. The website of each plan is included in the appendix.

**Table 1.** General information on benefit packages in 32 counties.

County/District	Maximum days perprescription	Number of diseases	Accepted times per year	Expert panel	Deductible (Yuan)	Co-payment rate	Maximum ceiling (Yuan)	Start time
Cangxian	?	10	1	?	100	50%	500	Jan. 2008
Dongguang*	30	10	1	?	?	?	1000	Sep. 2007
Donghai	14	10	2	Yes	?	?	?	?
Fuchuan	15	14	2	Yes	0	100%	600	Apr. 2008
Jianghai	?	10	4	?	1000	30%	3000	Jan. 2008
Linzi	?	12	2	?	500	?	?	2009
Shouguang#	10	10	4	?	?	40–60%	1500	Jan. 2008
Zhao’an	?	7	?	No	0	35%	10000	Jan. 2009
Baodaojiang	?	11	1	No	500	20–35%	3000	?
Changzhi	30	25	12	Yes	0	70%	5000	Jan. 2009
Chongren	30	11	4	Yes	100	60%	1000	Feb. 2009
Huichun	?	10	?	?	300	20–50%	1000	Jan. 2006
Huaibin*	?	10	1	Yes	0	50–70%	600	Jul. 2009
Jinxi	30	13	?	?	300	In	1000	Jul. 2006
Qianjiang	?	9	1	No	?	?	900	Nov. 2009
Tianmen#	30	16	1	Yes	0	80%	1000	Jan. 2009
Wangjiang	60	25	?	?	?	?	?	Mar. 2009
Yuquan	?	17	?	Yes	0	?	?	2008
Yuexi*	?	28	?	?	0	40%	3000	Jan. 2008
Zezhou	?	20	?	Yes	?	30%	5000	Jan. 2008
Dianjiang	?	12	?	?	50	40%	500	Jan. 2007
Gangcha	?	17	?	?	0	30–50%	600	?
Gaoling	?	12	?	Yes	?	40–50%	1500	Dec. 2006
Jiangyou#	?	9	?	No	?	In×0.8	2000	Sep. 2008
Liangzhou	20	6	Varies	?	1000	30%	3000	Oct. 2007
Nanjian*	?	9	?	No	0	50%	1000	Dec. 2008
Ningshan*	?	15	1	Yes	?	50%	2500	Aug. 2009
Qingchuan	?	13	?	Yes	?	30%	3000	Jan. 2008
Shiqian*	28	22	1	?	?	50%	1200	Mar. 2008
Su’nan	7	4	1	Yes	500	30%	2000	Jan. 2009
Tongzi	20	23	?	Yes	In	In	45000	Mar. 2009
Wansheng	15	8	1	Yes	0	50%	500	Aug. 2007

(1) *Denotes poverty-stricken county; # Denotes rich county.

(2) “In” refers to inpatient reimbursement rates.

(3) The counties in the middle of the table are in the central region, those at the top are in eastern China, those at the bottom are in western China.

(4) ? Denotes that the NRCMS County Office did not provide the relative information.

### Analytical framework and classification of diseases

**Figure 1.**
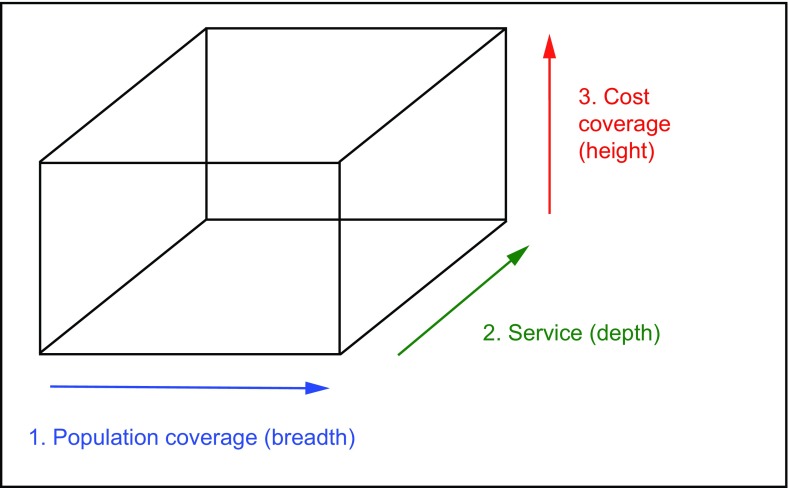
The three-dimensional universal coverage model ^22^.

The research used the Busse *et al.* conceptual framework to study coverage decisions as published by the World Bank ^22^. This is based on the idea that a benefit package is decided by coverage and benefit entitlements, including breadth (population coverage), depth (service coverage), and height (cost of coverage). This can be represented by a three-dimensional coverage model ( Figure 1).

This paper compares the different plans according to the three dimensions of coverage. We recorded the main points of every plan including reimbursement scope, chronic diseases selected, the deductible line, co-payment ratio and ceiling, methods of cost control, whether an expert panel had been created, and how many times enrolment of beneficiaries is carried out per year. We have attempted to determine the reasoning behind the different packages and produce recommendations for how it should operate. We classified the types of diseases covered according to the ICD-10.

## Results

### Who is “Entitled”?

Concerned that the number of beneficiaries could get out of control, the NRCMS offices have put a very strict procedure in place to avoid “moral hazard”. As with all BPCDO, one cannot be a beneficiary unless two conditions are met: the first is that they are a member of the NRCMS; the second is that they have a chronic disease. The first stipulation is easy to authenticate, but for the second, the counties differ in the methods used to judge if a person has a chronic disease or not.

Typically, there are two methods of judgment. One is where the NRCMS office empowers a county-level hospital to make the decision, usually the People’s Hospital in the applicant’s county, although some counties use different county-level hospitals according to the different townships. Another method, more often used, is where the NRCMS office uses a specifically created expert panel made up of delegates from different county-level hospitals (usually the People’s Hospital, Hospital of Traditional Chinese Medicine, Maternity & Child Care Centre etc.) to assess whether patients have a chronic disease. The NRCMS office convenes the experts to decide who should be eligible beneficiaries; generally, different diseases have different experts assigned to make the decision. Once a patient becomes a beneficiary, he/she can receive subsidies in the following years, although they must undergo a regular audit to check that they have continued membership of the NRCMS. Out of our 32 research counties, 14 counties had set up expert panels for this purpose. Of the remaining counties, eight counties had not set up a panel and the other counties did not mention whether they had an expert panel.

The current procedure to be designated as a beneficiary is very complicated. The applicant has to get certification from his/her village community first, and then go to a township hospital to confirm the basic health information in person, and submit a form with certification that he/she is suffering from at least one type of chronic disease. Certification must be provided by a county-level hospital or higher (Municipal hospital or Provincial hospital) if the applicant has been an inpatient. If the applicant was not an inpatient, they must provide evidence that they are suffering from at least one chronic disease from a level higher than a county hospital.

Sometimes, evidence of the severity of the disease must be provided by the applicant. For example, hypertension in phase III (ICD I10.XO5) will receive a subsidy, but not in phase II (ICD I10.X04), so the applicant has to show evidence that his/her condition is serious and should be in phase III. The township hospital will collect the information provided by the applicant and transfer it to the county NRCMS Office. The latter will then decide whether the applicant becomes a beneficiary. It takes a long time for a patient to become a beneficiary as the roll is updated slowly; of our 32 research counties, one county updated their beneficiary roll every month, three counties did this four times per year, three counties did this bi-annually, and ten counties did this annually. The other 15 counties did not mention how often they updated their beneficiary roll.

### What is covered?

***Selection of diseases.*** The chronic diseases listed in each BPCDO vary from between 4 and 28 diseases. In total, 41 types of chronic diseases are included in the 32 BPCDO, the frequency of inclusion of each type of chronic disease is listed in Table 2. No single chronic disease has been selected by all benefit packages, and some types of common chronic diseases, like coronary heart disease and chronic obstructive pulmonary disease (COPD), were only selected by under half the counties. Some counties have also selected endemic conditions such as sequelae of earthquake injuries and thalassemia.

**Table 2.** Selection of chronic diseases, ICD code and positive listing for reimbursement in 32 counties.

Disease	ICD code	Number of counties	Disease	ICD code	Number of counties
Diabetes mellitus	E10-E14	30	Fibrosis and cirrhosis of liver	K74	27
Hypertensive disease	I10-I15	27	Chronic renal failure	N17-N19	26
Sequelae of cerebrovascular disease	I69	26	Pulmonary heart disease	I26-I27	20
Schizophrenia	F20	20	Systemic lupus erythematosus	M32	19
Aplastic anemia	D60-D64	18	Tuberculosis (Active)	A15-A19	18
Atherosclerotic heart disease	I25.1	17	Rheumatoid arthritis	M05-M06	17
Failure and rejection of transplanted organs and tissues	T86	16	Leukemia	C90-C95	15
Chronic rheumatic heart disease	I05-I09	14	Chronic nephritic syndrome	N03	13
Heart failure	I50	10	Hyperthyroidism	E05	10
Cardiomyopathy	I42	7	Parkinson’s disease	G20	7
Chronic obstructive pulmonary disease	J44	6	Epilepsy	G40	5
Hemophilia	D66-D67	5	Chronic bronchitis	J41-J42	5
Chronic viral hepatitis	B18	4	Chronic active hepatitis	K71.5,K73.2	4
Inflammatory diseases of female pelvic organs	N70-N77	4	Hepatolenticular degeneration	E83.0	3
Protrusion of lumbar vertebraldisc	M51.0	3	Psoriasis	L40	2
Gout	M10	2	Chronic pancreatitis	K86.1	2
Peptic ulcer	K27	2	Simple goitre	E04.0	1
Thalassemia	D56	1	Leprosy	A30	1
Thrombangiitisobliterans	I73.1	1	Sequelae of earthquake injuries	T90-T94	1
Chronic cholecystitis	K81.1	1	Purpura and other haemorrhagic conditions	D69	1
Schistosomiasis	B65	1			

*Thirteen counties reimbursement for hypertension in phase ll, 6 counties in phase lll, others did not specify.

***Drugs and tests.*** Most counties listed some drugs and tests in their BPCDO. Some counties specified the scope of prescription according to disease, some even specified a detailed drug list for each disease, and some counties only prescribed within the Catalogue of Basic Medicines of the State, while others did not mention the scope of prescription. Of the 32 counties, 15 stipulated a maximum number of days for each prescription which varied from between 7 and 60 days. In addition, doctors are asked by the NRCMS office to prescribe in duplicate, so one copy is to be left at the hospital and the other copy is to be forwarded to the NRCMS office as evidence of reimbursement.

***Hospital choice.*** Some counties demand that beneficiaries must be treated within their county’s hospital, whereas others demand that special diseases must be treated at approved hospitals only. For example, mental disorders must be treated in the county mental hospital otherwise there can be no reimbursement. No county reimburses patients treated at a village clinic.

### How much cost-sharing is provided?

To control the cost of reimbursement, a co-payment rate, deductible line and ceiling have been set up by many counties.

***Deductible line and ceiling.*** Four counties have set a deductible line between 100 and 1000 Yuan, five counties have no deductible line, and others have not mentioned it. As for the ceiling, 15 counties have set a ceiling for reimbursement at 500 to 45000 Yuan depending on the disease. Two of the counties have set the ceiling in conjunction with inpatient expenses.

***Co-payment rate.*** The co-payment rate for most counties is between 60% and 80%, and is applicable for all types of chronic diseases. Some counties stipulate rates according to the disease and the level of the hospital, as different diseases have different reimbursement rates.

***Reimbursement frequency.*** Although inpatients can receive reimbursement as soon as they leave hospital, outpatients with a chronic disease are treated differently. Of the 32 counties, only four counties provide immediate reimbursement. Eight counties stated that they provide reimbursements once per quarter; five indicated that reimbursement is issued biannually, and twelve counties indicated that reimbursements are only issued annually. The other three counties did not mention how often they provide reimbursements.

Table 3 lists the generosity of cost-sharing within 32 BPCDO according to the number of selected chronic diseases, deductible line, reimbursement percentage, ceiling, the maximum days per prescription and the reimbursement frequency.

**Table 3.** Comparison of the generosity of cost-sharing within the 32 benefit packages for outpatient chronic disease treatments (Y = Yuan Renminbing).

	Number of counties	The least generous	mean	median	The most generous
Types of selected chronic diseases	32	4	13.5	11.5	28
Deductible line	21	1000 Y	271.5 Y	0 Y	0 Y
Reimbursement rate	26	20%	55%	50%	100%
Ceiling	28	500 Y	3719 Y	1100 Y	45000 Y
Maximum days of prescription	14	7	24	20	60
Reimbursement frequency per year	30	1		2	on the spot

## Discussion

### High mortality diseases and those with a heavy burden of disease should be included in the priority list

As mentioned in the analysis framework, the population coverage, service coverage, and cost coverage, can be represented as a cube. If any of the sides change, the shape of the cube changes too. Since it is impossible to allow infinity for any of the three dimensions, the BPCDO needs to balance the three factors. Among the three factors, disease selection is the core component as it directly influences the coverage of the population and the service coverage.

Our study shows that chronic diseases included in BPCDO vary, not only in number, but also in type. It is unreasonable to assume that the NRCMS fund could include all chronic diseases, so there needs to be an objective criterion for choosing the types of disease for inclusion. We think that high-mortality diseases, and the heavier-burden diseases (based on DALYs) should be taken into account to determine priorities. According to the China Statistical Yearbook 2007, the top 10 causes-of-death in rural China are malignant tumors (24.8%), cerebral vascular disease (20.6%), diseases of the respiratory system (17.2%), heart disease (14.8%), injury and intoxication (9.0%), digestive system diseases (2.7%), endocrine nutritional/metabolic diseases (1.5%), diseases of the genitourinary system (1.2%), nervous system diseases (0.8%) and mental disorders (0.6%).

According to the disease surveillance points system in rural China in 2002 (which can be seen as the initial stage of the death registration system), the highest proportion of deaths due to chronic diseases are caused by cancers, cerebrovascular disease, COPD, ischemic heart disease, hypertensive disease, liver disease and tuberculosis ^23, 24^. We were not able to obtain detailed information regarding burden of diseases for rural China, but the World Health Organization listed the leading causes of chronic disease in 2004 as: (according to the percentage of total DALYs in sequence), lower respiratory infections, unipolar depressive disorders, ischemic heart disease, cerebrovascular disease, COPD, refractive errors, hearing loss, adult onset diabetes and diabetes mellitus ^25^. Our study found that most of the previously listed common diseases appeared in some BPCDO. However, there are still counties which have omitted very common chronic diseases, such as ischemic heart disease and COPD. From the national perspective, our opinion is that the cause of death diseases and the heavier burden diseases (DALYs) should be used as primary-priority-setting criteria.

From synthesis of the information of leading cause-of-death diseases in rural areas in 2005, and morbidity of chronic diseases in rural areas in 2008 (China Health Statistical Yearbook 2009), we think the following 21 types of chronic diseases should be included at a national level: hypertensive diseases, diabetes mellitus, COPD, malignant tumours, sequelae of cerebrovascular disease, pulmonary heart disease, schizophrenia, chronic rheumatic heart diseases, inflammatory diseases of female pelvic organs, chronic viral hepatitis, fibrosis and cirrhosis of liver, atherosclerotic heart disease, rheumatoid arthritis, chronic nephritic syndrome, leukemia, heart failure, chronic renal failure, cardiomyopathy, Parkinson’s disease, chronic active hepatitis, chronic bronchitis and epilepsy. Patients suffering from other types of chronic diseases should get aid from the Medical Financial Assistance Scheme, not from the NRCMS. From the province perspective, the list can be adjusted according to the local spectrum of disease, which county managers can implement. For each province, the causes of death and burden of diseases in rural areas requires further research.

### Comparison with inpatient benefit packages

The most obvious difference between inpatient and outpatient benefit packages is their reimbursement rate. The overall real reimbursement rate for inpatients was 15% in 2007, but only 4% for outpatients ^26^. Therefore, the introduction of BPCDO intends to increase the reimbursement for chronic disease outpatients.

Inpatient benefit packages were introduced at the same time as the NRCMS in 2003 and, with development over the past few years, have developed some common principles which must be adhered to. For example, all inpatient benefit packages have a deductible line and ceiling; the higher the hospital level, the higher the deductible line and the lower the co-payment rate. To some extent, these policies have achieved their goals to induce inpatients to the low-level hospitals, and encourage doctors to use less expensive and higher-quality drugs. However, the BPCDO was introduced later and is less specific. Our study found that some of the BPCDO do not mention their maximum days per prescription or how many times they update their beneficiary roll per year. Forty percent of BPCDO lacked a deductible line, ceiling or co-payment rate. Some BPCDO (which we searched but did not use) did not even list the types of diseases they would reimburse. These obscure BPCDO must create difficulties for the practitioners and patients.

### Comparison with the general outpatients benefit package

As well as the BPCDO, there are also benefit packages for general outpatients with reimbursement for minor illnesses. More than half of the counties have general outpatients benefit packages since the NRCMS was implemented. There are two types of general outpatients benefit packages. The Household Medical Saving Account (HMSA) is one of these packages and, as its name suggests, some of the insured contribution (for example, 7–15 Yuan per capita per year, accounts for 40–70% of the premium) is a compulsory deposit in the HMSA. This can then be used for both outpatient and inpatient medical expenses for anyone within the family. This type of benefits package is used more often in Western China which is economically underdeveloped (91% of western counties in 2004) ^27^ as the HMSA means a lower premium, attracting more people to join the NRCMS. However, in the east of China, only 33% of counties chose the HMSA in 2004 ^27^, the remaining counties which have a general outpatient reimbursement, chose another type of benefit package termed “risk pool”. Risk pools work in the following way: the total fund of the NRCMS is divided into an inpatients and outpatients fund, the outpatients account for about 10–20% of the total fund, and when an insured member sees a doctor as an outpatient they receive 20–30% reimbursement; however, the ceiling is only 50–200 Yuan per capita per year. There were 1% of counties in the west of China and 13% in the east that implemented a risk pool in 2004 ^27^. Some researchers revealed that the function of the HMSA is similar to private savings, which violates the underlying political principle of “cooperation” and cannot achieve the stated aim of “solidarity” in the NRCMS ^28^. The effect of reduced impoverishment by the HMSA is not as obvious as that of the risk pool ^29^, so there is a tendency for more and more counties to choose risk pools at the present ^30^.

Besides the differences mentioned above, there are three points of difference between the general outpatient benefits package and the BPCDO. Firstly, the general outpatient benefits package allows outpatients get reimbursement from a village clinic (only licensed clinics, normally one village has one licensed clinic), but the BPCDO does not. Secondly, general outpatients benefit packages reimburse patients immediately (the hospital cashier), but the beneficiaries of the BPCDO need to wait for several months and possibly up to a year. Thirdly, the ceiling and reimbursement rates for the general outpatients benefits package are far less than the BPCDO ^31^, which is the main reason why the BPCDO is separate from general outpatients benefits package. However, the percentage of funds that should be allocated to outpatients requires further research.

### It is necessary to introduce a uniform policy at the national level?

Saltman and Figueras argue that decentralized management in the health system is possibly beneficial for efficiency and immediate responses to new problems, but the experiences of many countries have demonstrated that major policy framework decisions in health systems should be made at the central level ^32^. Our research confirmed this theory and we found that the BPCDO display great differences between different counties. Another study also found substantial differences in NRCMS policy design between regions ^33^. Only 6 of the 32 BPCDO include all of the essential factors. Some counties’ policies appear reasonable, others do not, and some even include obvious errors. For example, patients who are suffering infectious pulmonary tuberculosis are eligible for free medical treatment at an infectious diseases hospital ^34^, but some of the BPCDO still include this condition in their lists. We think that uniform principles of BPCDOa at the national level might avoid some unanticipated mistakes. A national policy on BPCDO should at a minimum include eight key components: (1) a specific list of types of disease using the ICD classification; (2) a specified deductible line; (3) co-payment rate; (4) a reimbursement ceiling; (5) maximum days per prescription; (6) the process of becoming a beneficiary, such as what evidence patients should provide, and whether it is decided by an expert panel; (7) how many times the beneficiary roll is updated per year; (8) whether beneficiaries receive reimbursement immediately, and if not, when they will receive it. In addition, the percentage of the chronic disease outpatient’s fund in relation to the total NRCMS fund should be specified.

### Limitations

Methodologically, our study had two potential limitations. All of the plans came from websites which may lead to “online bias” as not all counties announce their plans on the internet. We had difficulties in testing whether they carried out their plans according to their written documents. Secondly, the omission of counties with incomplete information on their websites might have led us to depict a more positive picture than exists in reality.

## Conclusions

A comparison of the BPCDO in 32 counties in the NRCMS shows substantial differences in the quality of the BPCDO and the level of reimbursement between counties. There is no gold standard for choosing the types of diseases for inclusion in the BPCDO, so we think it is imperative that a principle for the reimbursement of chronic disease should be set up at a national level. Any national BPCDO should include at least 8 essential factors, and 21 types of chronic diseases for the whole country to be covered.

## Note added in proof

Sadly, on 24 April 2013 Chuangzhou Xu, PhD passed away aged 38 following an acute myocardial infarction. He had dedicated his professional career as a health economist to improving healthcare in rural China through his research and teaching at the Northwest A&F University in Shaanxi, the University of Adelaide, and Shaanxi Normal University. Chuangzhou would have hoped that this publication contributed to this cause by highlighting persisting large inequities in rural healthcare in China, not only between the urban and rural health insurance schemes but also between rural regions.
