# Supplementary material for: Benefit packages for chronic disease outpatients in the New Rural Cooperative Medical Scheme in 32 Chinese counties
Source: F1000Res. 2013 Jun 10;2:137. [Version 1] doi: 10.12688/f1000research.2-137.v1 (PMC3829132; doi:10.12688/f1000research.2-137.v1)
Supplement: Peer review history

# Peer review: Referee response for version 1

**Published:** 2013-09-10

**Authors:** Qunhong Wu

**Affiliations:** 1 Department of Social Medicine, School of Public Health, Harbin Medical University, Harbin, 150081, China

1) The title of the paper is clear and outlines key information in this article. 

2)The abstract has provided a concise and fairly good summary. 

3) The description on the study design, method as well as the analysis of the findings are comprehensive.

 

4) The conclusions are justified based on the results. 

5) The limitation of the data has been clearly stated within the article. As many of the descriptions are based on the design instead of the implications of the results, some finding of relevant studies should be cited within the discussion to provide more evidence and to support the conclusion and suggestions. 

Despite this I would like to 'Approve' this article as it has provided a very comprehensive review on the NCMS's benefit package designs for NCD outpatients in China.

I have read this submission. I believe that I have an appropriate level of expertise to confirm that it is of an acceptable scientific standard.

# Peer review: Referee response for version 1

**Published:** 2013-07-04

**Authors:** M. Rashad Massoud

**Affiliations:** 1 Department of Public Health, University Research Co LLC, Bethesda, MD, USA

The article is interesting in that it compares chronic conditions benefits package in 32 counties in China. It uses a framework based on three dimensions of coverage: population, service and cost. It shows differences between counties and concludes with the necessity to standardize these across counties.

I have read this submission. I believe that I have an appropriate level of expertise to confirm that it is of an acceptable scientific standard.
